# Radiocontrast-Diatrozate and Iopromide-Induced Severe Hypocalcemia Leading to Painful Tetanic and Severe Laryngeal Spasm: A Near Fatal Encounter

**DOI:** 10.7759/cureus.26999

**Published:** 2022-07-18

**Authors:** Janhavi Mahajan, Abhinav Kadam, Prerna Verma, Sunil Kumar, Sourya Acharya

**Affiliations:** 1 Department of Medicine, Jawaharlal Nehru Medical College, Datta Meghe Institute of Medical Sciences (Deemed to be University), Wardha, IND

**Keywords:** loperamide, diatrozate, laryngealspasm, tetany, hypocalcemia

## Abstract

Routinely advised radiological investigations like contrast-enhanced scans can have adverse effects on electrolyte imbalance due to the administration of Radiocontrast. Urograffin (Diatrizoate) and Ultravist (Iopromide) are some of the common examples of contrasts given to patients that possibly led to chelation of the calcium in serum causing severe carpopedal spasms and painful muscular tetany in our patient. Here we report a case of a 32-year-old female patient with acute gastroenteritis who presented with complaints of pain in the abdomen, multiple episodes of vomiting, and loose stools underwent contrast-enhanced computed tomography (CECT) scan of the abdomen, and developed severe painful muscular tetanic spasms after intravenous and oral contrast was administered.

## Introduction

Tetany is a condition marked by increased neuromuscular excitability produced by a variety of metabolic disorders. The clinical presentation range from asymptomatic individuals to life-threatening situations, regardless of the causal reasons [1]. Adult stridor is most commonly caused by abscesses or swelling of the upper airway, malignancies, and paralysis or dysfunction of the vocal cords. Hypocalcemia-induced laryngospasm is a rare cause of stridor. One of the possible side effects is contrast media-related nephropathy, generally expressed as serum creatinine levels and urine creatinine clearance. However, contrast media can have different renal toxic effects on the renal tubular cells other than these two as an imbalance in electrolyte metabolism [1,2].

We give here the instance of a 32-year-old female patient who had severe carpopedal spasms and painful muscular tetany after experiencing abdominal pain, vomiting, and loose stools.

## Case presentation

A 32-year-old female patient presented to the hospital with chief complaints of pain in the abdomen, tenderness in the epigastrium, vomiting, and loose motions for 1 week which was followed by dark-colored stools one day ago. She had no history of diabetes mellitus, hypertension, thyroid disorder, or bronchial asthma. On examination, pulse rate was 100/min, regular in rhythm. BP was 100/60 mmHG in the right arm in the supine position and Respiratory rate was 20 breaths/min. The rest of the general examination was unremarkable.

On systemic examination there was bilateral air entry present in all lobes of the lungs, heart sounds were normally heard and the abdomen was found to be soft and non-tender. All the routine investigation reports are shown in Table 1. Serum calcium was found to be 9 mEq/L on admission, while magnesium value was 1.7 mEq/L on admission. Her serum lipase levels were 230 U/L and her serum amylase level was 90 U/L. Her serum parathormone (PTH) level was 40 pg/mL.

**Table 1:** Basic laboratory investigation report of the patient

Parameters	Before administration of contrast	After administration of contrast	
Hemoglobin (gm%)	11.9	13.8	
Mean Corpuscular Hemoglobin Concentration (in g/L)	34.4	32.1	
Mean Corpuscular Volume (in fl)	89.9	90.1	
Mean Corpuscular Hemoglobin (in pg)	30.9	28.9	
Total Red Blood Cell count (x 10^6^/L)	3.85	4.78	
Total White Blood Cell count (x 10^3^/L)	4300	6300	
Total platelet count (x 10^12^/L)	1.81	2.05	
Hematocrit (%)	34.6	43	
Monocytes	04	03	
Granulocytes	65	65	
Lymphocytes	30	30	
Red Cell Distribution Width	13.6	14	
Liver function test			
Alkaline phosphatase (u/L)	81	105	
Alkaline transferase (u/L)	7	23	
Aspartame transferase (u/L)	15	28	
Total proteins (g/L)	6.1	7.3	
Albumin (g/L)	3.3	4.2	
Total bilirubin (uMol/L)	0.5	0.6	
Bilirubin conjugated (uMol/L)	0.3	0.3	
Bilirubin unconjugated (uMol/L)	0.2	0.3	
Globulin (g/L)	2.8	3.1	
Renal function test			
Urea (mg/dl)	11	16	
Creatinine (mg/dl)	0.7	0.6	
Sodium (mEq/L)	137	138	
Potassium (mEq/L)	3.3	4.9	
Serum electrolytes			
	Immediately after administration of radio-contrast		
Calcium (mEq/L)	3.9	10.3	
Magnesium (mEq/L)	0.9	2.2	
Phosphate (mg/dl)	3.5	4.0	
Vitamin D levels (ng/dl)	35	40	
Paratharmone level (pg/ml )	40	45.5	

An electrocardiogram showed normal sinus rhythm and no obvious abnormality was detected in the chest x-ray. Ultrasonography abdomen and pelvis was done which revealed mild edematous small bowel. The gastroenterologist's opinion was taken in view of persistent vomiting, loose stools, and abdominal pain and was advised to treat it as acute gastroenteritis. A contrast-enhanced computed tomography (CECT) of the abdomen was advised on the same day, as symptoms did not resolve. There was no evidence of acute pancreatitis on the CECT abdomen.

**Figure 1:**
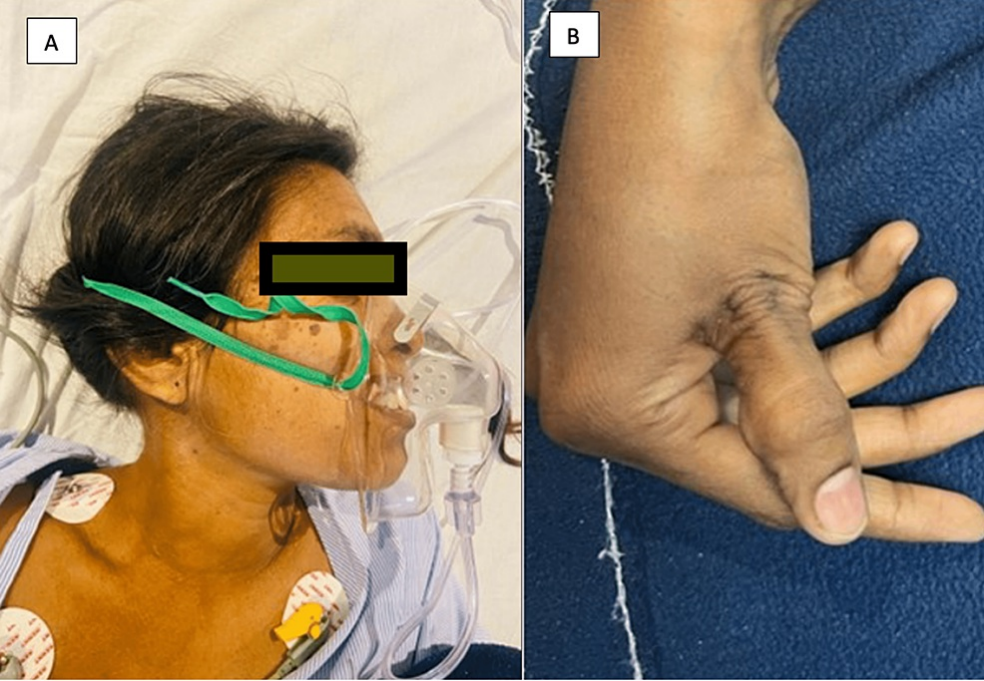
(A) Severe laryngospasm. (B) Carpopedal spasm.

Immediately after CECT, the patient developed tetanic contractions, severe carpopedal spasms (Figure 1A) over her fingers, and severe laryngospasm (Figure 1B) leading to stridor, for which she was shifted to Medicine Intensive care unit.

On examination, the Chvostek sign was found to be positive. Serum electrolytes were repeated immediately, which revealed severe hypocalcemia; serum calcium - 3.9 mg/dL, serum albumin - 3.3 mg/dL and corrected calcium level 4.5 mg/dL and severe hypomagnesemia; serum magnesium - 0.9 mg/dL. Urinary calcium was found to be normal ruling out urinary calcium loss. Injection calcium gluconate infusion of 10 meq diluted in 10 mL normal saline given intravenously over 10 minutes every four hours and Injection magnesium sulfate 2 g intravenously over 10 minutes every four hourly was administered. Serum electrolytes were repeated where hypocalcemia and hypomagnesemia resolved, restoring Ca value to 10.3 and Mg value to 2.2 PO_4_ value to 3.5 mg/dL. In due course of hospital stay, the patient’s condition improved and was discharged in stable condition with follow-up advised within seven days or as needed.

## Discussion

Calcium (Ca++) is a multivalent cation, which plays an important role in cellular functions. Confusion, tingling, laryngospasm, stridor, muscular spasms, numbness in the hands, feet, and face, depression, hallucinations, muscle aches, brittle nails, and an increased risk of bone fractures are all symptoms of hypocalcemia [1]. The Chvostek and Trousseau signs are also an important feature of severe hypocalcemia, which were positive in our case as well.

The majority of delayed adverse reactions to radiographic contrast media are rash, skin redness, and skin swelling. Nausea, vomiting, and dizziness have also been reported after the administration of the contrast agent. These reactions are typically mild and non-life threatening and resolved on their own so they are frequently overlooked by the treating physician. The mechanism of action of contrast-induced electrolyte imbalance, brief hypocalcemia, is to be confirmed yet. Although not a single case has been reported with a similar presentation and the cause is uncertain but in our case, it is likely due to the chelation of calcium due to the sodium calcium edetate (E385) present in the diatrizoate [2]. The reduction in Calcium levels that we observed seems to be largely attributable to the chelating agents and, to a lesser extent, to hemodilution from the added volume and hyperosmolarity of these solutions or to their high ionic strength [2,3].

Hypocalcemia is a rare phenomenon in patients in which radio contrasts are administered. Stridor is most commonly seen in children due to nutritional deficiency of calcium but is rare in adults due to various calcium homeostasis mechanisms [3]. The presence of stridor and laryngospasm in our adult patient due to hypocalcemia after possible chelation of calcium due to radiocontrast injection is a quiet rare scenario and could be the cause of this acute life-threatening emergency [4]. Contrast media that do not contain calcium chelating activity cause less disturbances in systemic calcium metabolism and the use of such media might be safer for certain patients as in this case.

## Conclusions

In case of an emergency like laryngospasm, electrolyte analysis of magnesium and calcium might be helpful in the diagnosis and treatment of the patients. Hypocalcemia should be considered in the differential diagnosis of stridor/laryngospasm at the onset for the primary as well as tertiary care physician. Additionally, the patients must be followed for complications such as hypocalcemia after injection of radiocontrast agents specially Urograffin (Diatrizoate) and Ultravist (Iopromide) which has chelating properties.
